# Knowledge and Awareness of Ocular Allergy Among the General Population of Makkah, Saudi Arabia: A Cross-Sectional Study

**DOI:** 10.7759/cureus.87947

**Published:** 2025-07-14

**Authors:** Hassan Alqurashi, Yousef M Alzahrani, Fahad Alzahrani, Omar M Alqurashi, Salh F Alanizi, Rawan Alzahrani, Mokhtar M Shatla

**Affiliations:** 1 Preventive Medicine, Makkah Health Cluster, Makkah, SAU; 2 Family Medicine, King Fahad Armed Forces Hospital - Jeddah, Jeddah, SAU; 3 Medical Affairs, King Fahad Armed Forces Hospital - Jeddah, Jeddah, SAU; 4 Medicine and Surgery, Umm Al-Qura University, Makkah, SAU; 5 College of Medicine, Umm Al-Qura University, Makkah, SAU

**Keywords:** allergic conjunctivitis, health literacy, ocular allergy, public health, saudi arabia

## Abstract

Background

Ocular allergies represent a growing public health concern, yet awareness and knowledge gaps persist - particularly in high-risk regions like Makkah, Saudi Arabia, with its unique environmental allergens. This study assessed ocular allergy knowledge among Makkah’s general population.

Methods

A cross-sectional, questionnaire-based study was conducted with 404 adults (aged 18-60 years), recruited via convenience sampling. An Arabic-language Google Forms (Google Inc., Mountain View, CA, USA) questionnaire evaluated knowledge across eight domains: definition, symptoms, anatomy, prevention, treatment, and risk factors. Data were analyzed using IBM SPSS Statistics for Windows, Version 27 (released 2020; IBM Corp., Armonk, NY, USA), along with the Mann-Whitney U and Kruskal-Wallis tests.

Results

The mean knowledge score was 3.41/8 (SD = 1.92), indicating limited awareness. Key gaps included treatment options (30% correct) and affected eye anatomy (34.2% correct). Females scored higher than males (3.73 vs. 2.93, p < 0.001), and university-educated participants outperformed others (3.92 vs. 2.75-3.00, p < 0.001). Only 16.8% achieved high knowledge (six to eight correct answers).

Conclusions

Significant knowledge deficits exist, particularly regarding treatment and disease mechanisms. Targeted interventions should prioritize at-risk groups (males and less-educated individuals) through community and digital health campaigns.

## Introduction

Ocular allergy, though a common condition, represents a significant public health concern due to its increasing prevalence and impact on quality of life [1]. The condition, which is often underestimated, arises from a hypersensitivity reaction triggered by exposure to allergens, leading to immune-mediated inflammation of the conjunctiva [2]. Globally, nearly 40% of the population experiences ocular allergy at some point in their lives, with higher prevalence rates observed in developing regions such as Latin America, Asia, and West Africa [1,3,4]. In Saudi Arabia, environmental factors such as dust, pollen, and extreme temperatures may exacerbate the burden of ocular allergies [5,6], particularly in urban areas like Makkah, where allergen exposure is high due to its arid climate and high population density. Despite this, there is a lack of data on the prevalence and public awareness of ocular allergy in Makkah, underscoring the need for targeted research.

Ocular allergies encompass a spectrum of conditions, including seasonal allergic conjunctivitis (SAC), perennial allergic conjunctivitis (PAC), vernal keratoconjunctivitis (VKC), atopic keratoconjunctivitis (AKC), and giant papillary conjunctivitis (GPC) [7]. SAC and PAC, the most common forms, are typically driven by environmental allergens such as pollen, dust mites, and pet dander, manifesting with symptoms like itching, redness, tearing, and eyelid swelling [8]. In contrast, VKC and AKC are more severe, chronic forms that can lead to corneal complications and visual impairment if left untreated [3,4]. The burden of these conditions is particularly pronounced among children and adolescents, with many cases first emerging during these formative years [9]. However, ocular allergies can affect individuals of all ages, necessitating broad public health strategies to mitigate their impact.

The management of ocular allergies relies on accurate diagnosis, allergen avoidance, and appropriate pharmacotherapy [10]. Topical antihistamines, mast cell stabilizers, and corticosteroids are commonly employed, though their use must be carefully balanced against potential side effects, such as elevated intraocular pressure in glaucoma patients or cataract formation [1,11]. Over-the-counter (OTC) medications provide relief for mild cases, but severe or refractory symptoms often require multidisciplinary care involving allergists and ophthalmologists [1,10]. Despite the availability of treatments, gaps in public knowledge about ocular allergies may lead to underdiagnosis, mismanagement, or delayed care, exacerbating the condition's morbidity.

In Makkah, the unique combination of environmental allergens, high population density, and a lack of prior studies on ocular allergy awareness creates a critical gap in public health knowledge. Understanding the level of awareness and knowledge among the general population is essential for designing effective educational campaigns and improving clinical outcomes. This study aims to assess the awareness and knowledge of ocular allergies among the general population of Makkah, Saudi Arabia. By identifying gaps in public understanding, the findings could inform targeted interventions to enhance disease recognition, promote timely treatment, and reduce the burden of ocular allergies in the region.

## Materials and methods

Study design 

This study utilized a cross-sectional questionnaire-based design to evaluate the knowledge and awareness of ocular allergies among the general population of Makkah, Saudi Arabia. 

Population

The target population comprises adults aged 18 to 60 years who reside in Makkah, ensuring a representative sample of the city’s diverse demographic. A convenience sampling technique was employed to recruit participants, as this method was practical for reaching a broad audience within the study’s timeframe. The inclusion criteria focused on adults (aged 18 and above) living in Makkah, while individuals outside the specified age range or non-residents were excluded to maintain the study’s relevance and accuracy.

Sample size calculation

The sample size was determined using OpenEpi version 3.0, a statistical tool designed for epidemiological studies. The calculation was based on Makkah’s estimated population of 10,991,006, with a 95% confidence interval and a 5% margin of error. This yielded a minimum required sample size of 385 participants. To account for potential incomplete or unusable responses, the target sample size was increased to 404 participants, ensuring robust and reliable data for analysis.

Data collection tool

A structured, self-administered online questionnaire was developed using Google Forms (Google Inc., Mountain View, CA, USA) to facilitate easy distribution and data collection. The questionnaire was made available in Arabic, the primary language of the target population, to ensure comprehension and accessibility (see Appendix). The instrument was adapted from validated tools used in prior studies on ocular allergies, with modifications to suit the local context. It consisted of two main sections: the first section collected data on age, gender, and educational level to categorize and analyze responses based on these variables. The second section included eight items designed to evaluate participants’ understanding of ocular allergies, covering topics such as definition, symptoms, affected eye anatomy, prevention methods, treatment options, and risk factors.

The questionnaire was reviewed by experts in Ophthalmology and Public Health to ensure its validity and relevance. Pilot testing was conducted with a small group to identify and address any ambiguities or technical issues before full deployment.

Questionnaire distribution

The survey link was disseminated through popular social media platforms, including Twitter, WhatsApp, and Instagram, as well as local community networks in Makkah. This approach was chosen to maximize reach and engagement among the target population. Participants were provided with an electronic informed consent form, which outlined the study’s purpose, confidentiality measures, and the voluntary nature of participation. Only those who consented were allowed to proceed with the survey.

Statistical analysis

All statistical analyses were performed using IBM SPSS Statistics for Windows, Version 27 (released 2020; IBM Corp., Armonk, NY, USA). Initially, descriptive statistics were generated to summarize the characteristics of the participant sample. Frequencies and percentages were calculated for categorical demographic variables, including gender, age group, and educational level. Descriptive statistics, including frequencies and percentages, were also calculated for the individual knowledge and awareness items, identifying the proportion of correct responses for each item. The total knowledge score, calculated by summing the correct responses across the eight knowledge items (range: 0-8), was summarized using descriptive statistics, including mean and standard deviation. The total knowledge score was further categorized into three ordinal levels (Low: 0-2 correct; Medium: 3-5 correct; and High: 6-8 correct), and the frequency distribution across these levels was determined and visualized using a bar chart.

To examine differences in the total knowledge score based on demographic characteristics, the Kolmogorov-Smirnov test was conducted to detect the nature of the knowledge score distribution. A Mann-Whitney U test was conducted to compare total knowledge scores between male and female participants. Kruskal-Wallis H tests were used to compare total knowledge scores across different categories of age groups (15-25, 26-40, and >40 years) and educational level (Primary, Intermediate, Secondary, and University Degree). For any significant Kruskal-Wallis test results, Bonferroni post hoc comparisons were conducted to identify specific group differences. Statistical significance for all inferential tests was determined using an alpha level of p < 0.05 and p < 0.01.

Ethical considerations

Ethical approval for the study was obtained from the Institutional Review Board (IRB) of Umm Al-Qura University, Makkah, Saudi Arabia, ensuring compliance with ethical standards for human research (approval no. HAPO-02-K-012-2024-10-2297). Participants were fully informed about the study’s objectives, their rights, and the measures taken to protect their confidentiality. No personally identifiable information was collected, and participation was entirely voluntary. These steps were taken to uphold ethical principles and foster trust among participants.

## Results

A total of 404 participants from the Makkah region of Saudi Arabia were included in the study. The majority of participants were female (n = 241, 59.7%), while male participants constituted 40.3% (n = 163) of the sample. Regarding age distribution, the largest group fell within the 26-40 years age range (n = 161, 39.9%), followed closely by those aged over 40 years (n = 151, 37.4%). Participants aged 15-25 years represented the smallest age group (n = 92, 22.8%). The educational level distribution indicated that nearly half of the participants held a university degree (n = 197, 48.8%). Other educational levels included secondary (n = 91, 22.5%), intermediate (n = 64, 15.8%), and primary (n = 52, 12.9%) (Table 1).

**Table 1:** Descriptive Statistics of Participant Demographics (N = 404)

Variable	Category	N	%
Gender	Female	241	59.7
	Male	163	40.3
Age Group	15-25 years	92	22.8
	26-40 years	161	39.9
	> 40 years	151	37.4
Educational Level	Primary	52	12.9
	Intermediate	64	15.8
	Secondary	91	22.5
	University Degree	197	48.8

Table 2 presents the distribution of responses for each specific knowledge and awareness item regarding ocular allergy. Participant knowledge varied considerably across the items. The highest proportion of correct answers was observed for the item assessing awareness of the condition's commonness (55.2% correct), closely followed by recognizing itching as a key symptom (54.5% correct). Moderate levels of knowledge were demonstrated regarding the best method for prevention (45.3% correct) and smoking as a risk factor (45.0% correct). Participants showed lower levels of knowledge concerning the basic definition of ocular allergy (40.3% correct), the specific parts of the eye affected (34.2% correct), particularly the transmissibility of the condition (36.1% correct), and the medications used for treatment (30.0% correct). The mean total knowledge score, compiled from these eight items, was 3.41 (SD = 1.92) out of a maximum possible score of 8, suggesting a generally limited level of knowledge among the study participants.

**Table 2:** Frequency Distribution of Correct and Incorrect Answers for Ocular Allergy Knowledge Items Note: N represents the frequency of participants, and % represents the percentage of participants. Correct answers score 1; incorrect or unknown answers score 0.

Knowledge Item (Question Text)	Item Focus	Correct Answer, N (%)	Incorrect Answer, N (%)
What is your understanding of Ocular Allergy?	Definition	163 (40.3%)	241 (59.7%)
Itching is the first symptom for most people with ocular allergy	Key Symptom	220 (54.5%)	184 (45.5%)
The parts of the eye affected by ocular allergy include	Affected Anatomy	138 (34.2%)	266 (65.8%)
Can ocular allergy be transmitted from person to person?	Transmissibility	146 (36.1%)	258 (63.9%)
Ocular allergy is one of the most common conditions people generally suffer from.	Prevalence/Commonness	223 (55.2%)	181 (44.8%)
What is the best way to prevent ocular allergy?	Prevention	183 (45.3%)	221 (54.7%)
What medications are used to treat ocular allergy?	Treatment Options	121 (30.0%)	283 (70.0%)
Smoking is a risk factor for developing ocular allergy.	Risk Factors	182 (45.0%)	222 (55.0%)
Overall Knowledge Score (Range 0-8)	Mean ± SD	3.41 ± 1.92	

**Figure 1:**
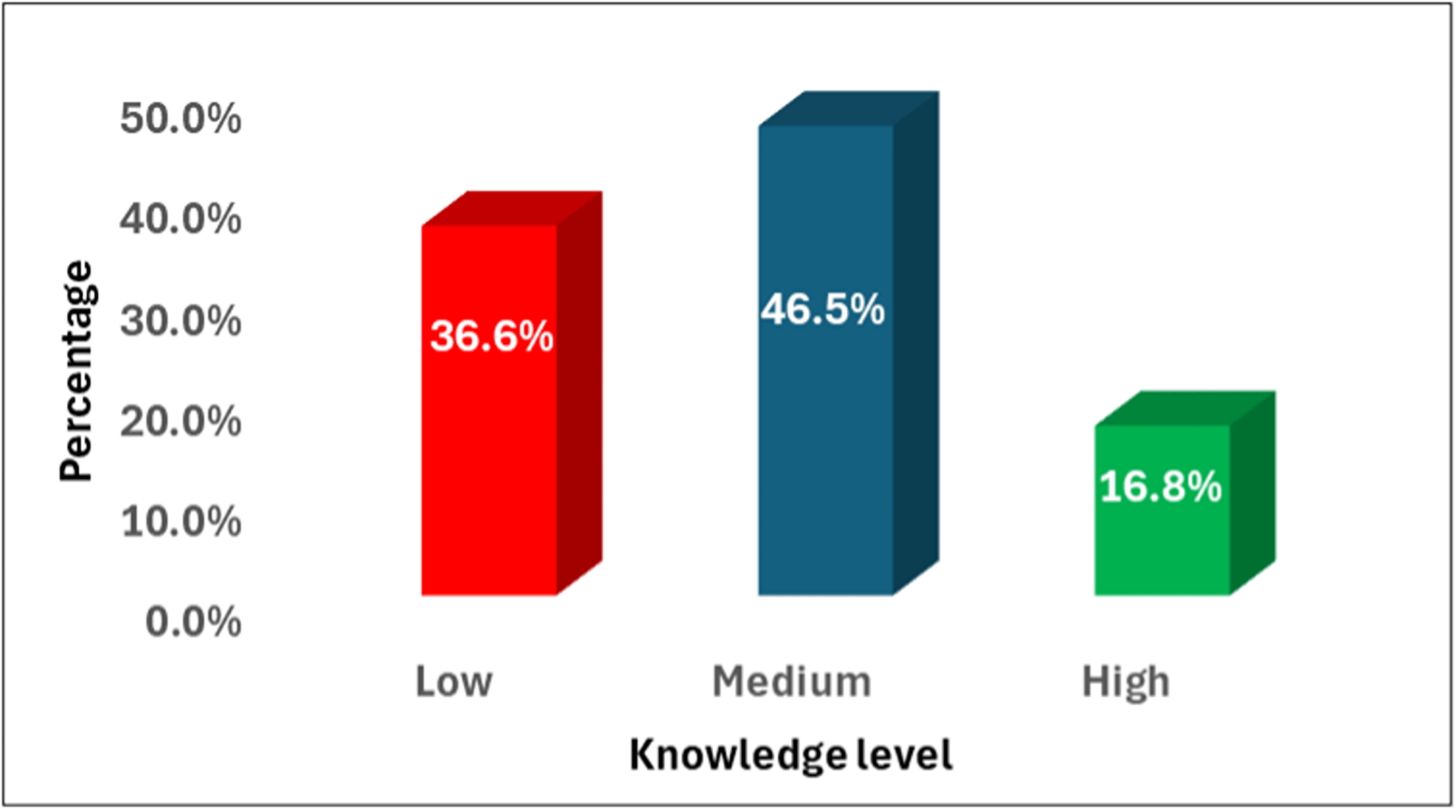
Distribution of Ocular Allergy Knowledge Levels Across Participants

In addition, Figure 1 illustrates the distribution of ocular allergy knowledge levels across participants. The largest proportion of the sample demonstrated a medium level of knowledge (46.5%), representing participants who answered between 3 and 5 of the 8 knowledge items correctly. Participants with a low level of knowledge (scoring 0-2 correct answers) comprised a significant portion of the sample (36.6%). Only a minority of participants (16.8%) were categorized as having a high level of knowledge (scoring 6-8 correct answers). 

As shown in Table 3, female participants (M = 3.73, SD = 2.04, mean rank = 219.81) demonstrated a significantly higher mean knowledge score compared to male participants (M = 2.93, SD = 1.63, mean rank = 176.91) (p < 0.001). Moreover, subsequent Bonferroni post hoc comparisons indicated that participants aged 26-40 years (M = 3.80, SD = 2.12) had significantly higher knowledge scores than both the 15-25 years age group (M = 2.98, SD = 1.85, p = 0.003) and the over 40 years age group (M = 3.25, SD = 1.66, p = 0.035) (Table 4).

**Table 3:** Comparison of Ocular Allergy Knowledge Score by Demographic Characteristics **Significant at p < 0.01 M = Mean, SD = Standard Deviation, U = Mann-Whitney U statistic, Z = Z-score, H = Kruskal-Wallis H statistic

Variable	Category	M ± SD	Mean Rank	Test Statistic	p-value
Gender	Female	3.73 ± 2.04	219.81	U = 15470	< 0.001**
	Male	2.93 ± 1.63	176.91	Z = -3.675	
Age	15-25 years	2.98 ± 1.85	176.58	H = 10.078	0.006**
	26-40 years	3.80 ± 2.12	222.83		
	> 40 years	3.25 ± 1.66	196.62		
Educational Level	Primary	2.75 ± 1.49	162.42	H = 23.543	< 0.001**
	Intermediate	3.00 ± 1.62	180.83		
	Secondary	2.95 ± 1.55	180.25		
	University	3.92 ± 2.12	230.4		

**Table 4:** Bonferroni Post-Hoc Comparisons for Age Group Differences *Significant at p < 0.05 and **Significant at p < 0.01; All p > 0.05, adjusted for multiple comparisons. SE = Standard Error

Age Group	Age Group	Mean Difference	SE	p
15-25 years	26-40 years	-0.817	0.248	0.003**
	> 40 years	-0.273	0.251	0.828
26-40 years	> 40 years	0.543	0.215	0.035*

Lastly, Table 5 indicates that participants with a university degree had significantly higher knowledge scores than participants with primary (p < 0.001), intermediate (p = 0.004), and secondary (p < 0.001) educational levels. There were no statistically significant differences (p > 0.05) in knowledge scores among the primary, intermediate, and secondary education groups.

**Table 5:** Bonferroni Post-Hoc Comparisons for Educational Level Differences *Significant at p < 0.05 and **Significant at p < 0.01; All p > 0.05, adjusted for multiple comparisons. SE = Standard Error

Educational Level	Educational Level	Mean Difference	SE	p
Primary	Intermediate	-0.250	0.347	0.999
	Secondary	-0.195	0.323	0.999
	University Degree	-1.174*	0.29	< 0.001**
Intermediate	Secondary	0.055	0.303	0.999
	University Degree	-0.924*	0.267	< 0.001**
Secondary	University Degree	-0.979*	0.235	< 0.001**

## Discussion

The findings of this study provide critical insights into the knowledge and awareness of ocular allergies among the general population of Makkah, Saudi Arabia. The results reveal significant gaps in public understanding, with an overall mean knowledge score of 3.41 out of 8, indicating limited awareness.

The study identified notable participants’ limited understanding of ocular allergies, particularly regarding disease definition (40.3% correct), affected eye anatomy (34.2% correct), and treatment options (30.0% correct). These findings align with prior research in other regions. For instance, a study in Ghana reported that only 34.7% of participants could correctly identify ocular allergy symptoms, while a survey in India found that 45% of respondents misunderstood the condition's non-communicable nature [12,13]. The consistency of these results across diverse populations underscores a global challenge in public health education about ocular allergies. However, our study contrasts with research from high-income countries like the United States of America (USA), where awareness levels are higher. Meltzer et al. [2] found that 62% of American participants accurately identified common ocular allergy treatments, attributing this to widespread access to healthcare information and targeted advertising of OTC medications. This disparity highlights the influence of socioeconomic and healthcare infrastructure differences on health literacy, where individuals with lower socioeconomic status - including lower educational attainment and income - tend to have lower health literacy, and a well-developed healthcare infrastructure, with easily understandable health information resources such as pamphlets, websites, and trained healthcare professionals, leads to higher health literacy [14-16].

Female participants in our study demonstrated significantly higher knowledge scores (M = 3.73) compared to males (M = 2.93), a trend agreeing with other studies in Oman and Egypt [17,18]. This may reflect gender-based disparities in healthcare engagement, as women are more likely to seek medical advice for allergic conditions and participate in health-related surveys [19]. Cultural factors in Saudi Arabia, where women often manage family health, could further explain this gap. However, women in Arab countries were found to have a higher prevalence of ocular diseases, and cultural norms and societal expectations were suggested to hinder women's access to healthcare, reduce their health literacy, and limit their decision-making power regarding their eye health [20].

Participants aged 26-40 years scored significantly higher than younger (15-25 years) and older (>40 years) groups. This aligns with findings by Villegas et al. [21], which linked peak knowledge to middle adulthood, a period of increased health responsibility. The lower scores among older adults may reflect generational gaps in health education or cognitive decline, while younger participants’ limited awareness could stem from infrequent healthcare utilization for non-severe conditions, as supported by other previous studies elsewhere [22-24].

University-educated individuals outperformed those with lower education levels, consistent with evidence linking education to health literacy, especially regarding conjunctivitis [25]. A Brazilian study by Guedes et al. [7] similarly found that higher education correlated with better allergy management knowledge. This underscores the need for tailored interventions for less-educated populations, such as community-based workshops or simplified educational materials.

Over a third (36.1%) of participants erroneously believed ocular allergies are contagious, mirroring findings in other studies [12,13,26]. This misconception may delay treatment-seeking, as patients fear stigmatization. Public campaigns clarifying the immune-mediated nature of allergies are recommended in Makkah, as they have been shown to be effective elsewhere [27]. Only 30% knew about standard treatments (e.g., antihistamines), compared to 58% in a Spanish study [25]. This gap may reflect limited pharmacist engagement in Saudi Arabia, where pharmacies are primary sources of OTC medication advice but often lack structured allergy education programs.

While 45% recognized smoking as a risk factor, this was lower than the 67% reported in the USA [2]. Cultural norms around smoking in Saudi Arabia, where male smoking rates are high (30%-40%), may increase this risk. Makkah’s arid climate and high pollen/dust levels exacerbate ocular allergies [1,28], yet awareness remains low. Similar challenges were noted in other countries with similar climates, especially in the Middle East region [27,29], where desert climates increased allergy prevalence but public knowledge lagged behind. Unique to Saudi Arabia, pilgrimage-related overcrowding in Makkah may amplify allergen exposure, necessitating targeted education for residents and visitors, as a combination of crowded conditions, dust, and allergens introduced by pilgrims from different parts of the world can increase the risk of allergic reactions and respiratory illnesses during the Hajj.

To address the significant knowledge gaps identified in this study, a multi-pronged approach to public health interventions is recommended. First, community education programs should be implemented through collaboration with mosques, schools, and primary care centers, as these institutions serve as trusted sources of information within the Makkah community. The success of Kenya's community health worker program in improving eye disease knowledge and community-based eye care services provides a valuable model for such grassroots educational initiatives [30,31]. Second, enhancing pharmacist training programs could substantially improve public understanding of ocular allergies, particularly regarding treatment options. The Portuguese experience demonstrates that well-trained pharmacists can increase patient adherence to allergy treatments by 40% [7,32], suggesting this could be an effective strategy in the Saudi context. Third, digital campaigns leveraging social media platforms should be prioritized, given their effectiveness in reaching broad audiences [33]. The UAE's success in using Instagram reels to increase allergy knowledge by 25% among young adults offers a replicable model for Makkah’s tech-savvy population. Finally, integrating ocular health modules into school curricula could yield long-term benefits, as evidenced by Australia's National Allergy Strategy, which reduced childhood allergy complications by 30% [34]. These interventions should be culturally adapted to ensure relevance and effectiveness within the Saudi context.

While these findings provide valuable insights, some limitations must be acknowledged. The use of convenience sampling may limit the generalizability of the results to the broader Makkah population, and the reliance on self-reported data introduces potential response bias. Future research should address these limitations by employing random sampling techniques and incorporating clinical validation of participants’ knowledge. Longitudinal studies would be particularly valuable for assessing the long-term impact of the proposed interventions and tracking changes in public awareness over time. Additionally, qualitative research exploring cultural perceptions of ocular allergies could provide deeper insights into the barriers to knowledge acquisition and help tailor interventions more effectively. By addressing these research gaps, while implementing the recommended public health strategies, significant progress can be made in improving ocular allergy awareness and management in Makkah and similar regions.

## Conclusions

In conclusion, this study reveals significant gaps in ocular allergy knowledge among Makkah’s population, with particularly low awareness of treatment options and disease mechanisms. Demographic disparities highlight the need for targeted interventions, especially for males, older adults, and less-educated individuals. The findings align with global trends but underscore unique regional challenges, including environmental allergens and healthcare access barriers. Effective strategies should combine community education, pharmacist training, digital campaigns, and school-based programs, adapted to Saudi Arabia’s cultural context. This study provides a foundation for future research using randomized designs and longitudinal assessments. Addressing these knowledge gaps through tailored public health initiatives could reduce ocular allergy morbidity and improve the quality of life for affected individuals in Makkah and similar regions.

## Appendices

(1) What is your understanding of Ocular Allergy? (Knowledge)

■ Correct Answer (Score 1): التهاب العين استجابة لمادة غريبة غير ضارة (Eye inflammation in response to a harmless foreign substance)

■ Incorrect Answers (Score 0): All other options (Discomfort and pain inside the eye, resulting from frequent eye rubbing, I don't know)

(2) Itching is the first symptom for most people with ocular allergy. True/False/I don't know? (Awareness)

■ Correct Answer (Score 1): صحيح (True)

■ Incorrect Answers (Score 0): خطأ (False), لا اعلم (I don't know)

(3) The parts of the eye affected by ocular allergy include: (Knowledge)

■ Correct Answer (Score 1): الملتحمة، القرنية والجفون (Conjunctiva, Cornea, and Eyelids) - This is the most comprehensive and accurate answer among the options, as ocular allergy can affect all these parts (e.g., allergic blepharitis, keratoconjunctivitis).

■ Incorrect Answers (Score 0): All other options (Cornea, Conjunctiva, Conjunctiva and Cornea, I don't know)

(4) Can ocular allergy be transmitted from person to person? True/False/I don't know? (Knowledge)

■ Correct Answer (Score 1): خطأ (False) - Ocular allergy is an immune response, not an infection.

■ Incorrect Answers (Score 0): صحيح (True), لا اعلم (I don't know)

(5) What is the best way to prevent ocular allergy? (Awareness)

■ Correct Answer (Score 1): الابتعاد عن المصادر التي تحفز حدوث حساسية العين (Avoiding sources that trigger ocular allergy) - This is the primary preventive measure.

■ Incorrect Answers (Score 0): All other options (Using anti-allergy medications - this is treatment/symptom management, Visiting ophthalmology clinics - this is seeking care, Not getting close to people who have eye allergy - irrelevant as it's not contagious).

(6) What medications are used to treat ocular allergy? (Knowledge)

■ Correct Answer (Score 1): مضادات الهيستامين (Antihistamines) - These are a primary class of medication for ocular allergy.

■ Incorrect Answers (Score 0): All other options (Warm compresses, Antibiotics - for infections, Lubricating eye drops - supportive care but not primary anti-allergic medication, I don't know)

(7) Smoking is a risk factor for developing ocular allergy. True/False/I don't know? (Knowledge)

■ Correct Answer (Score 1): صحيح (True) - Smoking is an irritant and can worsen or contribute to allergic conditions.

■ Incorrect Answers (Score 0): خطأ (False), لا اعلم (I don't know)

(8) (Awareness question included in Knowledge score) Ocular allergy is one of the most common conditions people generally suffer from. True/False/I don't know? (Awareness)

■ Correct Answer (Score 1): صحيح (True) - As stated in your introduction (40% global prevalence), it is a very common condition.

■ Incorrect Answers (Score 0): خطأ (False), لا اعلم (I don't know)
